# Limits in reliability of leg-spring and joint stiffness measures during single-leg hopping within a sled-based system

**DOI:** 10.1371/journal.pone.0225664

**Published:** 2019-12-05

**Authors:** David Diggin, Ross Anderson, Andrew J. Harrison

**Affiliations:** 1 Department of Exercise Science and Athletic Training, Ithaca College, Ithaca, New York, United States of America; 2 Biomechanics Research Unit, Department of Physical Education and Sport Sciences, University of Limerick, Limerick, Ireland; Universitat Konstanz, GERMANY

## Abstract

Research examining the reliability of stiffness measures during hopping has shown strong consistency in leg-spring stiffness (k_leg_), but high variability in joint stiffness (k_joint_) measures. Sled-based systems (SBS) reduce movement degrees-of-freedom and are used to examine stretch-shortening cycle (SSC) function under controlled conditions. The aim of this study was to examine the reliability of k_leg_ and k_joint_ during single-leg hopping within an SBSKinematic and kinetic data were collected on four occasions (Day_1, Day_2, Day_3 and Day_3_Offset_). Participants completed two trials of single-leg hopping at different frequencies (1.5, 2.2 and 3.0 Hz) while attached to an inclined-SBS. Stiffness was determined using models of leg-spring (k_leg_) and torsional (k_joint_) stiffness. Statistical analysis identified absolute and relative measures of reliability. Results showed moderate reliability for k_leg_ at 1.5 Hz between inter-day testing bouts, and weak consistency at 2.2 and 3.0 Hz. Examination of intra-day comparisons showed weak agreement for repeated measures of k_leg_ at 1.5 and 2.2 Hz, but moderate agreement at 3.0 Hz. Limits in k_leg_ reliability were accompanied by weak-to-moderate agreement in k_joint_ measures across inter- and intra-day testing bouts. Results showed limits in the reliability of stiffness measures relative to previous reports on overground hopping. Lack of consistency in k_leg_ and k_joint_ may be due to the novelty of hopping within the current inclined-SBS. Constraints imposed on the hopping task resulting from SBS design (e.g. additional chair mass, restricting upper body movement) may have also influenced limits in k_leg_ and k_joint_ reliability. Researchers should consider these findings when employing inclined-SBS of a similar design to examine SSC function.

## Introduction

Stretch-shortening cycle (SSC) tasks are characterised by eccentric lengthening immediately followed by concentric shortening [1]. These actions are typical of human movement (e.g. walking, running, jumping and throwing) and serve to enhance concentric force output and movement efficiency [1,2]. Knowledge of mechanisms controlling SSC function are therefore relevant to general health and athletic performance. SSC function has subsequently been examined during walking [3], running [4,5], jumping [4], under fatigued conditions [6–10] and before and after training [11,12].

It is understood that the efficiency of SSC task performance is regulated by a combination of elastic and neural mechanisms [1]. Of importance to elastic mechanisms of SSC performance is the property stiffness (k). Stiffness represents the resistance of a body to deformation and reflects the elastic nature of the body [13,14]. In humans, greater stiffness is found to enhance the operational effectiveness of the SSC [15–17], yet higher levels of stiffness also contribute to musculoskeletal injury [18]. When examining stiffness characteristics during SSC tasks, researchers often model the elastic nature of the entire leg as a linear-spring (i.e. k_leg_) [9,19–22]. Torsional stiffness models are also used to clarify the contribution of the stiffness of each joint (k_joint_) to k_leg_ [7].

The reliability of k_leg_ and k_joint_ measures has been examined during SSC tasks [22,23]. During double- and single-leg hopping, Joseph *et al*. [23] and Diggin *et al*. [22] showed good agreement (ICC > 0.80) for repeated measures of k_leg_ when hopping frequency is controlled. In contrast, measurement consistency declined (ICC = 0.20–0.86) during running and hopping at self-selected frequencies [23]. Furthermore, k_joint_ measures have consistently demonstrated questionable reliability during natural hopping and running tasks. Joseph *et al*. [23] showed poor reliability for all k_joint_ variables measured (ICC = 0.51–0.92; CV > 20%). Diggin and colleagues [22] showed strong consistency for repeated measures of k_ankle_, but weak-to-moderate agreement for k_knee_ and k_hip_ (ICC = 0.57–0.93) during single-leg hopping. It must be noted that in efficient SSC tasks (e.g. hopping), participants must coordinate the body’s musculoskeletal structures (e.g. segments, muscles, joints) in an integrated fashion to achieve the movement goal. These requirements produce inherent variability in movement patterns which can impact movement consistency and measurement reliability [24]. It is possible that limits in k_leg_ and k_joint_ reliability previously reported, were caused by extraneous movements (esp. upper body) that impacted landing consistency.

Researchers have used sled-based systems (SBS) to offset issues associated with extraneous upper body movements [8,19,25,26]. While construction varies between laboratories, these systems often include rails positioned at angles below vertical orientation (e.g. 0°–30° to horizontal) [12,27]. A force plate mounted at the end of the system provides kinetic information of participants’ performance [6,25,28]. Participants perform SSC tasks (e.g. drop / rebound jumps, hopping) while attached to a chair / platform (sled) that slides up and down the inclined rails. SBS regulate levels of mechanical load experienced during SSC tasks by (i) altering rail angle [27]; (ii) employing a winch system to control initial eccentric loading [19,29]; and (iii) reducing movement degrees-of-freedom [10,30]. For lower body SBS, performance of SSC tasks while attached to the chair limits upper body movement and allows researchers to control impact velocities and eccentric loading [8,19]. Furthermore, the restricted path of the sledge chair might reduce task constraints and facilitate consistent landing patterns which can impact measurement reliability.

The reliability of kinematic and kinetic variables measured during SSC task performance within an SBS has been examined. Debenham and colleagues [10] reported strong agreement (ICC = 0.87) for ankle joint range-of-motion recorded during single-leg hopping tasks within an SBS. During drop (DJ) and rebound jump (RBJ) tasks, Flanagan and Harrison [19] reported strong consistency (Cronbach’s α > 0.95) for temporal and kinetic variables tested (e.g. flight time, F_z max_, reactive strength index). Similarly, during a simulated hopping task within an SBS, Furlong and Harrison [29] reported strong consistency (Cronbach’s α > 0.90) for temporal (CT, flight time) and kinetic (e.g. F_z max_, peak rate of force development) variables. Flanagan and Harrison [19] reported strong reliability (Cronbach’s α > 0.95) for repeated measures of k_leg_ during jumping tasks (Cronbach’s α > 0.95) within an SBS.

Given the purpose of the SBS is to reduce extraneous upper body movement to encourage landing and measurement consistency, it is possible that stiffness measures (esp. k_joint_) achieved during hopping within an SBS might demonstrate strong reliability. To date, the consistency of k_leg_ and k_joint_ measures recorded during hopping within an SBS has not been examined. The aim of this study was to examine the reliability of stiffness measures during hopping within an inclined-SBS. It was hypothesized that restricting extraneous upper body movement would facilitate strong consistency for k_leg_ and k_joint_ variables during hopping tasks.

## Materials and methods

### Participant characteristics

Thirty-two students (16 males and 16 females; mean ± SD age 21.3 ± 2.9 years; stature 1.70 ± 0.08 m; mass 69.9 ± 10.0 kg) volunteered to participate. Volunteers were recruited via e-mail circulated to the campus community. All participants were physically active as members of a university sports club, attending bi-weekly practices and represented the university at varsity level. Participants were injury free for at least six months prior to testing as determined via health screening questionnaire. Procedures were approved by the research ethics committee of the Faculty of Education and Health Science at the University of Limerick, and all participants provided written informed consent. To limit the effects of fatigue on stiffness measurement, participants were instructed to abstain from vigorous physical activity 24 hours before testing bouts.

### Experimental procedures

Participants completed a familiarization session one week prior to initial testing to acquaint them with procedures (Day_0) [31]. Following this, participants underwent testing on four occasions. The first (Day_1), second (Day_2) and third (Day_3) testing bouts took place at the same time of day, spaced three to seven days apart [23,32]. On the final test day, participants completed procedures twice; at their typically scheduled time (Day_3) and six hours prior to or following their scheduled time (Day_3_Offset_) [22].

**Fig 1:**
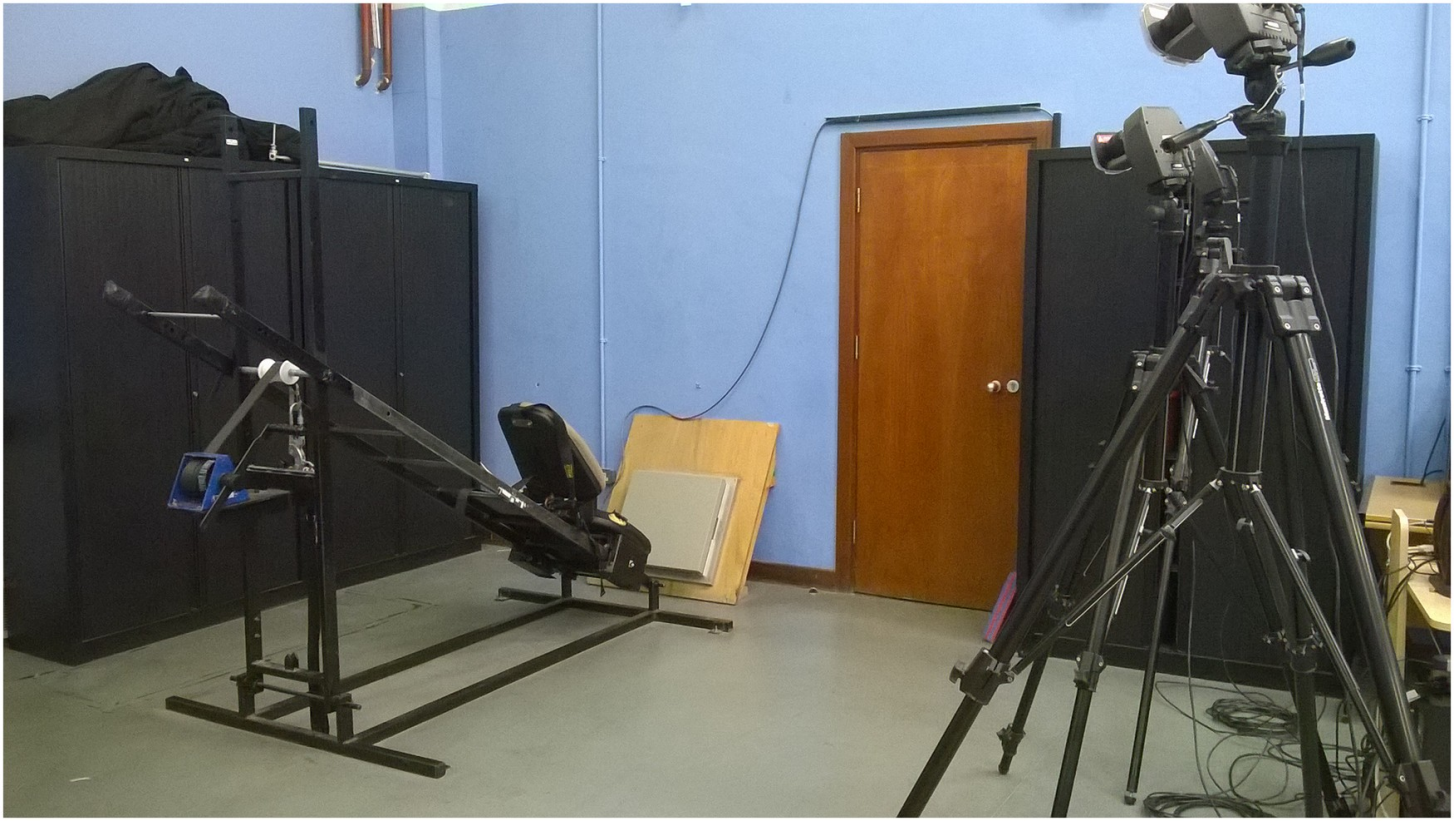
Image showing the experimental set-up for hopping trials within the sled-based system.

For each test, participants wore dark, tight-fitting clothing. Retro-reflective markers (14 mm) were placed at six anatomical locations on participants’ right side (acromion process, greater trochanter, lateral epicondyle of the femur, lateral malleolus, calcaneus and fifth metatarsal). Marker placement was conducted by the same tester throughout for consistency. During all test bouts (Day_0 –Day_3), participants completed two 10 second trials of single-leg hopping at each of 1.5, 2.2 and 3.0 Hz in time with a digital metronome (TempoPerfect Metronome, NCH Software, Greenwood Village, CO, USA) while secured within an SBS (Fig 1). The design of the SBS was described previously [12,19]. As previous research suggests little effect of leg dominance on k_leg_ or k_joint_ [23], participants were instructed to land as close as possible to the force plate center (AMTI OR6-5; AMTI, Watertown, MA, USA) in time with the metronome, using their right leg and natural hopping technique. Trials were accepted for analysis if participants hopped within ± 5% of the target frequency [33,34]. The order of hopping trials was randomly assigned, and participants received 60 seconds recovery between each trial to limit fatigue effects. An analogue triggering device was used to initiate 3D kinematic and kinetic data acquisition simultaneously. Kinematic data were recorded using three MAC Eagle cameras (MotionAnalysis Corporation, Santa Rosa CA, USA) operating at 200 Hz. Kinetic data were recorded at 1 kHz over the 10 second duration.

### Data processing

Analysis of data revealed an inconsistent delay in the initiation of kinematic and kinetic recordings. Whittlesey and Robertson [35] noted that kinematic and kinetic input signals must be temporally aligned to facilitate valid inverse dynamics analysis. Work in our laboratory has shown that maximum rate of change in fifth metatarsal marker acceleration profiles (i.e. peak jerk), coincide with ground contact to within 3.83 (± 1.06) ms of force plate criterion measures during overground hopping. Fifth metatarsal vertical coordinate data were subsequently differentiated to jerk [36]. The time of peak jerk was determined, and kinematic data were subsequently aligned with the time at which the vertical force increased above 5 N.

Marker trajectories were digitized using Cortex motion analysis software (version 2.1; MotionAnalysis Corporation, Santa Rosa, CA, USA). 3D kinematic and kinetic data were subsequently exported and analyzed using customized MS Excel macros. Recorded kinematic and kinetic data were concurrently filtered using a fourth-order Butterworth Low-Pass digital filter with an optimal cut-off of 11 Hz determined via residual analysis [36]. 3D coordinate data were subsequently interpolated to 1 kHz using a cubic spline. Filtered kinematic and kinetic data were used to calculate resultant joint moments occurring about ankle (M_ankle_), knee (M_knee_) and hip (M_hip_) joints throughout the ground contact phase of each trial using inverse dynamics [7,23,37]. Segment inertia and mass characteristics were determined using the standards of Dempster [38]. Having calculated resultant moments, average torsional stiffness of the ankle (k_ankle_), knee (k_knee_) and hip (k_hip_) were calculated as a ratio of changes in joint moment (ΔM_joint_) and angle (Δϴ_joint_) for respective lower limb joints [7,23].

In addition, k_leg_ was recorded throughout the ground contact phase of all hopping trials using the spring-mass model [21]. Thus k_leg_ was calculated as the ratio of F_z max_ and maximum leg compression (ΔLeg_L_) occurring during ground contact as measured from video records [39], where leg length represented the distance between the greater trochanter and the center-of-force [40]. In all analyzed trials, the temporal occurrence of discrete events of F_z max_ and ΔLeg_L_ coincided to within 10% of the hop period.

### Statistical analysis

The mean of 5 consecutive hops occurring within the middle of each 10-second trial was calculated for dependent variables and compared within and between experimental bouts. Differences between the means recorded for each experimental bout were established for each hopping frequency (HopFreq) using repeated measures ANOVA (parametric variables) or Friedman’s test (non-parametric variables). Reliability analysis employed both ‘absolute’ and ‘relative’ measures. A two-way random effects intra-class correlation coefficient (ICC) model examined the relative agreement between parametric and log-transformed dependent variables, while measurement consistency was established from effect size (ES) and ICC 95% confidence interval results [31,41,42]. Variables were considered strong when ICC ≥ 0.90 [43], the lower-bound level of the ICC 95% confidence interval (ICC_Lower_) > 0.80 [31] and Cohen’s ES < 0.50 (moderate effect) [44]. Moderate reliability occurred if ICC ranged from 0.80 to 0.89, ICC_Lower_ ≥ 0.70 and ES < 0.50, while ‘weak’ reliability occurred if the previous criteria were not met. Within-participant variation in dependent variables were also examined using typical error (TE) [41], however these statistics were not included in reliability indexing. All statistical analysis was conducted using SPSS (version 23.0, IBM Corporation, Armonk, NY, USA).

## Results

Results for control variables of contact time (CT; F _(1.3, 3.9)_ = 2.14; *p* > 0.05), flight time (FT; F _(1.7, 5.0)_ = 2.84; *p* > 0.05) and HopFreq (F _(1.8, 5.3)_ = 0.56; *p* > 0.05; Table 1) revealed no significant difference between means for these variables across inter-day testing bouts. Reliability analysis showed strong agreement for variables of CT and FT (ICC = 0.95–0.97; ICC_Lower_ = 0.81–0.92; ES = 0.01–0.09) at 1.5 and 3.0 Hz with moderate reliability evident at 2.2 Hz (ICC = 0.94; ICC_Lower_ = 0.79–0.92; ES = 0.10). Across intra-day testing bouts, analysis revealed no significant difference in CT (F _(2, 18)_ = 0.10; *p* > 0.05), FT (F _(2, 18)_ = 0.29; *p* > 0.05) and HopFreq (F _(1.6, 43.6)_ = 1.24; *p* > 0.05) when measured six-hours apart. The high ICC’s (0.92–0.98), moderate-to-high ICC_Lower_ coefficients (0.82–0.96) and low ES (0.04–0.13), suggest ‘strong’ intra-day agreement for variables of FT and CT. Despite moderate-to-strong reliability indices for FT and CT variables, HopFreq exhibited weak consistency throughout.

**Table 1:** Descriptive and reliability statistics (mean ± s) for control variables of CT, FT, and HopFreq recorded between inter- and intra-day testing bouts during hopping within a SBS.

	**Inter-day Comparisons**								
	**Mean (± s)**			**ICC**	**ICC 95% CI**		**TE**	**ES**	**Index**
**Variables**	**Day_1**	**Day_2**	**Day_3**		**Lower**	**Upper**			
**1.5 Hz**									
CT (s)	0.529 (0.063)	0.539 (0.052)	0.524 (0.060)	0.966	0.918	0.990	0.016	0.058	**Strong**
FT (s)	0.136 (0.064)	0.127 (0.054)	0.142 (0.060)	0.965	0.918	0.990	0.015	0.070	**Strong**
HopFreq (Hz)	1.50 (0.02)	1.50 (0.01)	1.50 (0.02)	0.681	0.468	0.830	0.02	0.12	Weak
**2.2 Hz**									
CT (s)	0.370 (0.029)	0.377 (0.025)	0.367 (0.020)	0.942	0.808	0.993	0.011	0.090	**Strong**
FT (s)	0.083 (0.027)	0.077 (0.026)	0.087 (0.021)	0.938	0.794	0.993	0.011	0.096	*Moderate*
HopFreq (Hz)	2.21 (0.03)	2.21 (0.02)	2.21 (0.02)	0.732	0.543	0.860	0.02	0.03	Weak
**3.0 Hz**									
CT (s)	0.274 (0.013)	0.274 (0.013)	0.274 (0.013)	0.949	0.872	0.987	0.006	0.006	**Strong**
FT (s)	0.059 (0.014)	0.059 (0.013)	0.060 (0.012)	0.948	0.868	0.987	0.006	0.032	**Strong**
HopFreq (Hz)	3.01 (0.04)	3.00 (0.03)	3.00 (0.03)	0.721	0.541	0.847	0.03	0.07	Weak
	**Intra-day Comparisons**								
		**Mean (± s)**		**ICC**	**ICC 95% CI**		**TE**	**ES**	**Index**
**Variables**		**Day_3**	**Day_3**_**Offset**_		**Lower**	**Upper**			
**1.5 Hz**									
CT (s)		0.524 (0.060)	0.516 (0.073)	0.970	0.935	0.988	0.014	0.080	**Strong**
FT (s)		0.142 (0.060)	0.151 (0.074)	0.969	0.935	0.988	0.011	0.086	**Strong**
HopFreq (Hz)		1.50 (0.02)	1.51 (0.02)	0.773	0.606	0.880	0.02	0.28	Weak
**2.2 Hz**									
CT (s)		0.367 (0.021)	0.360 (0.029)	0.981	0.959	0.993	0.007	0.083	**Strong**
FT (s)		0.087 (0.021)	0.093 (0.029)	0.982	0.961	0.994	0.007	0.077	**Strong**
HopFreq (Hz)		2.21 (0.02)	2.20 (0.03)	0.740	0.544	0.865	0.02	0.08	Weak
**3.0 Hz**									
CT (s)		0.274 (0.013)	0.275 (0.013)	0.921	0.827	0.970	0.006	0.131	**Strong**
FT (s)		0.06 (0.012)	0.059 (0.011)	0.916	0.818	0.969	0.007	0.044	**Strong**
HopFreq (Hz)		3.00 (0.03)	2.99 (0.04)	0.736	0.542	0.861	0.03	0.31	Weak

Abbreviations: CT = contact time; FT = flight time; HopFreq = hopping frequency; ICC = intraclass correlation coefficient; 95% CI = 95% confidence interval; TE = typical error; ES = effect size; Index = reliability index resulting from absolute and relative reliability statistics. Note. Central statistics represent the mean (± s) of five consecutive hops recorded for two trials across Day_1, Day_2, Day_3, and Day_3_Offset_ experimental bouts.

Friedman’s test revealed no significant change in k_leg_ (χ^2^ (5) = 0.68–10.23; *p* > 0.05) across Day_1, Day_2 and Day_3 testing bouts at any HopFreq. Despite exhibiting little change between days, reliability indices showed k_leg_ exhibited weak-to-moderate consistency (ICC = 0.71–0.89; ICC_Lower_ = 0.29–0.73; ES = 0.08–0.26) between inter-day bouts across all HopFreq (Table 2). Examination of kinematic and kinetic variables (Table 3) shows limits in k_leg_ measurement agreement originated from limits in F_z max_ and ΔLeg_L_ measurement consistency. Limits in k_leg_ measurement agreement, were accompanied by weak consistency in k_knee_ and k_hip_ between Day_1, Day_2 and Day_3 across all HopFreq (ICC = 0.62–0.84; ICC_Lower_ = 0.02–0.61; ES = 0.10–0.70). Only k_ankle_ exhibited moderate-to-strong consistency at slower HopFreq (ICC = 0.91–0.94; ICC_Lower_ = 0.76–0.83; ES = 0.17–0.18). Examination of kinematic and kinetic variables (Table 3) shows that despite limits to k_knee_ and k_hip_ at 1.5 Hz, participants exhibited weak-to-strong consistency for joint kinetic and kinematic variables. At 2.2 and 3.0 Hz, limits in k_knee_ and k_hip_ resulted from weak-to-moderate agreement for joint kinetics and kinematics. Data show that strong consistency in k_ankle_ measures at 1.5 Hz was accompanied by strong consistency in M_ankle max_ and Δϴ_ankle_. Limits in k_ankle_ measurement agreement at 2.2 and 3.0 Hz were accompanied by varied consistency in ankle kinematic and kinetic measures. Joint kinetics and kinematics showed no change (*p* > 0.05) between inter-day test bouts at any hopping frequency.

**Table 2:** Descriptive and reliability statistics (mean ± s) for variables of k_leg_ and k_joint_ recorded between inter- and intra-day testing bouts during hopping within a SBS.

	**Inter-day Comparisons**								
	**Mean (± s)**			**ICC**	**ICC 95% CI**		**TE**	**ES**	**Index**
**Variables**	**Day_1**	**Day_2**	**Day_3**		**Lower**	**Upper**			
**1.5 Hz**									
k_leg_ (kN.m^-1^)	4.85 (0.82)	4.70 (1.04)	5.00 (1.80)	0.887	0.731	0.968	0.57	0.08	*Moderate*
k_ankle_ (Nm.deg^-1^)	2.27 (0.69)	2.22 (0.69)	2.45 (0.64)	0.936	0.833	0.985	0.32	0.18	**Strong**
k_knee_ (Nm.deg^-1^)	1.14 (1.28)	1.65 (2.29)	0.94 (0.72)	0.789	0.485	0.940	7.44	0.10	Weak
k_hip_ (Nm.deg^-1^)	14.70 (14.18)	11.61 (5.16)	10.49 (5.17)	0.838	0.611	0.954	18.48	0.34	Weak
**2.2 Hz**									
k_leg_ (kN.m^-1^)	7.69 (1.25)	8.19 (1.63)	8.22 (1.29)	0.770	0.430	0.940	0.9	0.26	Weak
k_ankle_ (Nm.deg^-1^)	4.06 (1.57)	3.41 (0.93)	3.75 (1.08)	0.909	0.762	0.979	2.20	0.17	*Moderate*
k_knee_ (Nm.deg^-1^)	24.9 (31.56)	15.21 (12.57)	7.17 (6.22)	0.742	0.015	0.982	8.21	0.70	Weak
k_hip_ (Nm.deg^-1^)	109.77 (154.56)	100.68 (133.98)	121.02 (171.15)	0.623	0.376	0.794	17.37	0.05	Weak
**3.0 Hz**									
k_leg_ (kN.m^-1^)	11.5 (3.30)	11.01 (2.30)	12.79 (7.20)	0.713	0.288	0.925	5.11	0.20	Weak
k_ankle_ (Nm.deg^-1^)	5.12 (1.81)	4.53 (1.64)	5.04 (1.96)	0.884	0.695	0.973	1.69	0.03	Weak
k_knee_ (Nm.deg^-1^)	28.47 (27.18)	41.72 (74.19)	61.05 (82.54)	0.747	0.584	0.861	13.93	0.35	Weak
k_hip_ (Nm.deg^-1^)	47.84 (73.03)	20.57 (23.85)	34.73 (59.67)	0.625	0.382	0.795	17.30	0.17	Weak
	**Intra-day Comparisons**								
		**Mean (± s)**		**ICC**	**ICC 95% CI**		**TE**	**ES**	**Index**
**Variables**		**Day_3**	**Day_3**_**Offset**_		**Lower**	**Upper**			
**1.5 Hz**									
k_leg_ (kN.m^-1^)		5.00 (1.80)	5.00 (1.33)	0.858	0.699	0.944	1.05	0.11	Weak
k_ankle_ (Nm.deg^-1^)		2.45 (0.64)	2.61 (0.86)	0.833	0.635	0.937	0.76	0.13	Weak
k_knee_ (Nm.deg^-1^)		0.94 (0.72)	8.99 (8.98)	0.386	0.000	0.859	8.98	0.02	Weak
k_hip_ (Nm.deg^-1^)		10.49 (5.17)	17.93 (11.75)	0.864	0.694	0.951	10.85	0.18	Weak
**2.2 Hz**									
k_leg_ (kN.m^-1^)		8.22 (1.29)	11.76 (14.30)	0.594	0.091	0.854	1.68	0.35	Weak
k_ankle_ (Nm.deg^-1^)		3.75 (1.08)	5.39 (6.29)	0.847	0.642	0.948	1.13	0.25	Weak
k_knee_ (Nm.deg^-1^)		7.17 (6.22)	12.70 (12.48)	0.683	0.000	0.941	11.60	0.15	Weak
k_hip_ (Nm.deg^-1^)		121.02 (171.15)	24.71 (19.08)	0.888	0.417	0.997	16.18	0.10	Weak
**3.0 Hz**									
k_leg_ (kN.m^-1^)		12.79 (7.20)	12.56 (5.25)	0.865	0.705	0.949	3.90	0.17	*Moderate*
k_ankle_ (Nm.deg^-1^)		5.04 (1.96)	6.70 (4.14)	0.915	0.814	0.968	2.26	0.14	**Strong**
k_knee_ (Nm.deg^-1^)		61.05 (82.54)	6.76 (5.11)	0.287	0.000	0.747	7.95	0.50	Weak
k_hip_ (Nm.deg^-1^)		34.73 (59.67)	12.43 (11.61)	0.885	0.720	0.963	10.10	0.06	*Moderate*

Abbreviations: k_leg_ = leg-spring stiffness; k_ankle_, k_knee_, k_hip_ = stiffness variables for ankle, knee, and hip joints, respectively; ICC = intraclass correlation coefficient; 95% CI = 95% confidence interval; TE = typical error; ES = effect size; Index = reliability index resulting from absolute and relative reliability statistics. Note. Central statistics represent the mean (± s) of 5 consecutive hops recorded for 2 trials across Day_1, Day_2, Day_3, and Day_3_Offset_ experimental bouts.

**Table 3:** Descriptive (mean ± s) and reliability statistics for kinetic and kinematic variables recorded between inter-day testing bouts during hopping within a SBS.

	Inter-day Comparisons								
	Mean (± s)			ICC	ICC 95 % CI		TE	ES	Index
Variables	Day 1	Day 2	Day_3		Lower	Upper			
**1.5 Hz**									
F_z max_ (N)	435 (109)	423 (93)	446 (107)	0.976	0.943	0.993	23	0	**Strong**
M_ankle max_ (Nm)	53.27 (14.83)	52.83 (14.38)	54.72 (14.13)	0.936	0.846	0.981	6.11	0.07	**Strong**
M_knee max_ (Nm)	5.05 (10.89)	8.50 (7.76)	6.04 (9.57)	0.665	0.167	0.905	8.59	0.07	Weak
M_hip max_ (Nm)	75.35 (39.48)	84.88 (37.26)	94.19 (51.26)	0.953	0.888	0.986	14.69	0.29	**Strong**
Δ ϴ_ankle_ (°)	24.62 (5.67)	25.06 (5.66)	24.78 (5.47)	0.964	0.908	0.991	2.20	0.02	**Strong**
Δ ϴ_knee_ (°)	15.09 (6.92)	13.95 (6.46)	15.05 (5.93)	0.937	0.850	0.982	1.90	0.00	**Strong**
Δ ϴ_hip_ (°)	5.95 (3.20)	5.47 (2.95)	6.42 (3.05)	0.960	0.900	0.989	1.00	0.10	**Strong**
ΔLeg_L_ (m)	0.05 (0.05)	0.07 (0.04)	0.08 (0.05)	0.889	0.731	0.968	0.01	0.41	*Moderate*
**2.2 Hz**									
F_z max_ (N)	439 (82)	425 (77)	456 (66)	0.913	0.783	0.977	21	0	*Moderate*
M_ankle max_ (Nm)	53.77 (10.33)	50.53 (10.94)	54.11 (10.58)	0.847	0.589	0.965	6.01	0.02	Weak
M_knee max_ (Nm)	7.77 (5.04)	8.68 (7.37)	9.70 (8.68)	0.725	0.299	0.929	4.05	0.18	Weak
M_hip max_ (Nm)	75.40 (29.76)	67.25 (30.11)	89.95 (90.95)	0.842	0.585	0.963	46.03	0.19	Weak
Δ ϴ_ankle_ (°)	14.63 (3.03)	15.01 (3.03)	15.08 (2.41)	0.943	0.857	0.985	1.40	0.11	**Strong**
Δ ϴ_knee_ (°)	2.66 (1.25)	3.02 (1.32)	3.18 (1.30)	0.682	0.194	0.917	1.00	0.27	Weak
Δ ϴ_hip_ (°)	1.93 (1.11)	1.51 (0.88)	1.84 (0.99)	0.827	0.567	0.955	0.70	0.06	Weak
ΔLeg_L_ (m)	0.04 (0.03)	0.04 (0.03)	0.04 (0.03)	0.721	0.331	0.926	0.01	0.15	Weak
**3.0 Hz**									
F_z max_ (N)	446 (54)	445 (63)	458 (47)	0.967	0.917	0.991	16	0	**Strong**
M_ankle max_ (Nm)	53.39 (9.61)	54.40 (11.15)	60.71 (19.71)	0.922	0.807	0.980	4.70	0.36	**Strong**
M_knee max_ (Nm)	12.88 (11.33)	8.91 (11.13)	8.47 (12.46)	0.873	0.688	0.966	7.57	0.25	Weak
M_hip max_ (Nm)	49.92 (34.54)	51.79 (26.49)	71.57 (47.38)	0.883	0.712	0.969	15.89	0.40	*Moderate*
Δ ϴ_ankle_ (°)	10.93 (2.61)	12.49 (3.38)	11.87 (3.80)	0.901	0.755	0.974	2.00	0.19	*Moderate*
Δ ϴ_knee_ (°)	2.85 (1.04)	3.04 (1.82)	2.42 (2.11)	0.519	0.000	0.876	1.30	0.18	Weak
Δ ϴ_hip_ (°)	4.27 (1.81)	4.27 (2.03)	4.03 (1.58)	0.728	0.308	0.930	1.70	0.09	Weak
ΔLeg_L_ (m)	0.02 (0.02)	0.03 (0.02)	0.03 (0.02)	0.785	0.451	0.945	0.01	0.34	Weak

Abbreviations: ICC = intraclass correlation coefficient; 95% CI = 95% confidence interval; TE = typical error; ES = effect size; Index = reliability index resulting from absolute and relative reliability statistics; F_z max_ = maximum ground reaction force; M_ankle max_, M_knee max_, and M_hip max_ = maximum resultant moments recorded for ankle, knee, and hip joints, respectively; Δϴ_ankle_, Δϴ_knee_, and Δϴ_hip_ = relative angular displacement at ankle, knee, and hip joints, respectively; ΔLeg_L_ = change in leg length. Note. Central statistics represent the mean (± s) of 5 consecutive hops recorded for 2 trials across Day_1, Day_2, and Day_3 experimental bouts.

Examination of intra-day testing comparisons (Table 2) showed no change in k_leg_ between Day_3 and Day_3_Offset_ (χ^2^ (3) = 0.60–3.19; *p* > 0.05). Despite this, k_leg_ measures exhibited weak consistency at 1.5 and 2.2 Hz HopFreq (ICC = 0.59–0.86; ICC_Lower_ = 0.09–0.70; ES = 0.11–0.35) and moderate consistency at 3.0 Hz (ICC = 0.87; ICC_Lower_ = 0.71; ES = 0.17). Limits in k_leg_ reliability originated from strong and weak consistency in F_z max_ and ΔLeg_L_ respectively at all HopFreq. Repeated measure ANOVA results showed no change in k_joint_ variables between Day_3 and Day_3_Offset_ (F _(1.1, 22.6)_ = 0.17–1.07; *p* > 0.05). Despite this, k_knee_ and k_hip_ exhibited weak-to-moderate consistency between Day_3 and Day_3_Offset_ (ICC = 0.29–0.89; ICC_Lower_ = 0.00–0.72; ES = 0.02–0.50). In contrast, k_ankle_ exhibited strong consistency at 3.0 Hz but weak consistency at other hopping frequencies. Examination of kinematic and kinetic data (Table 4) showed limits in k_joint_ reliability originated from largely weak-to-moderate consistency in joint kinematic and kinetic inputs between Day_3 and Day_3_Offset_. Limits in consistency in kinematic and kinetic inputs occurred despite no change in these data between Day_3 and Day_3_Offset_ testing bouts (χ^2^ (3) = 0.98–3.86; *p* > 0.05).

**Table 4:** Descriptive (mean ± s) and reliability statistics for kinetic and kinematic variables recorded between intra-day testing bouts during hopping within a SBS.

	Intra-day Comparisons							
	Mean (± s)		ICC	ICC 95% CI		TE	ES	Index
Variables	Day_3	Day_3_Offset_		Lower	Upper			
**1.5 Hz**								
F_z max_ (N)	446 (107)	467 (125)	0.970	0.936	0.988	19	0	**Strong**
M_ankle max_ (Nm)	54.72 (14.13)	57.56 (18.48)	0.902	0.790	0.962	9.19	0.09	*Moderate*
M_knee max_ (Nm)	6.04 (9.57)	8.37 (14.59)	0.651	0.110	0.896	10.83	0.20	Weak
M_hip max_ (Nm)	94.19 (51.26)	84.64 (53.07)	0.852	0.679	0.944	19.04	0.05	Weak
Δ ϴ_ankle_ (°)	24.78 (5.47)	23.1 (5.0)	0.907	0.803	0.964	2.80	0.11	**Strong**
Δ ϴ_knee_ (°)	15.05 (5.93)	3.1 (6.8)	0.895	0.774	0.959	4.20	0.02	*Moderate*
Δ ϴ_hip_ (°)	6.42 (3.05)	5.2 (3.5)	0.953	0.899	0.981	0.90	0.06	**Strong**
ΔLeg_L_ (m)	0.08 (0.05)	0.09 (0.02)	0.857	0.696	0.944	0.01	0.13	Weak
**2.2 Hz**								
F_z max_ (N)	456 (66)	483 (83)	0.991	0.980	0.997	14	0	**Strong**
M_ankle max_ (Nm)	54.11 (10.58)	71.58 (51.69)	0.716	0.372	0.897	35.27	0.28	Weak
M_knee max_ (Nm)	9.70 (8.68)	10.93 (9.36)	0.752	0.414	0.919	7.36	0.10	Weak
M_hip max_ (Nm)	89.95 (90.95)	86.71 (71.85)	0.894	0.757	0.964	38.91	0.04	*Moderate*
Δ ϴ_ankle_ (°)	15.08 (2.41)	14.7 (5.0)	0.848	0.653	0.946	1.90	0.15	Weak
Δ ϴ_knee_ (°)	3.18 (1.30)	-2.3 (2.5)	0.885	0.744	0.959	1.40	0.23	*Moderate*
Δ ϴ_hip_ (°)	1.84 (0.99)	-1.9 (1.9)	0.833	0.621	0.940	0.80	0.23	Weak
ΔLeg_L_ (m)	0.04 (0.03)	0.06 (0.01)	0.643	0.171	0.878	0.01	0.23	Weak
**3.0 Hz**								
F_z max_ (N)	458 (47)	461 (54)	0.982	0.959	0.993	13	0	**Strong**
M_ankle max_ (Nm)	60.71 (19.71)	62.57 (16.98)	0.613	0.187	0.851	6.29	0.37	Weak
M_knee max_ (Nm)	8.47 (12.46)	10.59 (15.30)	0.804	0.519	0.937	9.30	0.22	Weak
M_hip max_ (Nm)	71.57 (47.38)	65.13 (49.60)	0.844	0.651	0.944	20.20	0.02	Weak
Δ ϴ_ankle_ (°)	11.87 (3.80)	9.50 (8.10)	0.975	0.946	0.991	5.30	0.19	**Strong**
Δ ϴ_knee_ (°)	2.42 (2.11)	-3.60 (2.50)	0.534	0.000	0.828	1.10	0.25	Weak
Δ ϴ_hip_ (°)	4.03 (1.58)	-5.10 (2.50)	0.799	0.563	0.924	2.00	0.34	Weak
ΔLeg_L_ (m)	0.03 (0.02)	0.04 (0.01)	0.749	0.435	0.913	0.01	0.01	Weak

Abbreviations: ICC = intraclass correlation coefficient; 95% CI = 95% confidence interval; TE = typical error; ES = effect size; Index = reliability index resulting from absolute and relative reliability statistics; F_z max_ = maximum ground reaction force; M_ankle max_, M_knee max_, and M_hip max_ = maximum resultant moments recorded for ankle, knee, and hip joints, respectively; Δϴ_ankle_, Δϴ_knee_, and Δϴ_hip_ = relative angular displacement at ankle, knee, and hip joints, respectively; ΔLeg_L_ = change in leg length. Note. Central statistics represent the mean (± s) of 5 consecutive hops recorded for 2 trials across Day_3 and Day_3_Offset_ experimental bouts.

## Discussion

SBS are used to examine SSC function under controlled conditions. Sled design regulates eccentric loading and limits extraneous movement to improve landing and measurement consistency. During overground hopping and running, k_leg_ has demonstrated good reliability [22,23]. In contrast, k_joint_ measures have demonstrated limited consistency. The reliability of k_leg_ and k_joint_ while hopping within an SBS had not been examined. This study provides a comprehensive analysis of the inter- and intra-day reliability of k_leg_ and k_joint_ achieved during hopping within an SBS.

Similar to the findings of Hobara and colleagues [21] k_leg_ and k_joint_ increased with increases in HopFreq in the present study. Values of 13.9–28.9 kN.m^-1^ have been reported for k_leg_ during natural hopping conditions on different surfaces and at different frequencies [22,23,30,45,46]. In addition, values of 6.9–12.0 Nm.deg^-1^ have been reported for k_ankle_ during natural hopping [45]. In the present study, stiffness values are lower than reported during natural hopping [22,23]. This can be explained by the orientation of the frame of the SBS (i.e. 30° to the horizontal) which reduces (half) the effect of gravitational acceleration [12,19]. Therefore, lower ground reaction forces (i.e. F_z max_) are expected in the present study relative to published reports on overground hopping. This is supported by the work of Harrison *et al*. [25] and Flanagan and Harrison [19] who reported lower values for k_leg_ during DJ and RBJ tasks (3.4–8.5 kN.m^-1^) performed within the current SBS, relative to natural conditions. The stiffness data recorded during the present study are in line with previous data reported for SSC tasks performed within an inclined-SBS.

Hobara *et al*. [21] showed increases in k_leg_ with increases in HopFreq. Thus, regulation of HopFreq between testing bouts was important to reliability analysis. In the current study HopFreq displayed weak-to-moderate consistency across all trials despite CT and FT variables showing strong agreement at all HopFreq between Day_3 and Day_3_Offset_. Inter-day comparisons showed strong consistency for CT throughout. In contrast, FT exhibited strong agreement at 1.5 and 3.0 Hz but moderate consistency at 2.2 Hz. Hopkins and colleagues [41] suggested low ICC’s occur in situations where between participant variation is low for a given variable. In the present study, HopFreq was regulated to within 5% of the target frequency. Thus, low levels of variability may have produced spurious reliability indices in HopFreq and in FT measures during hopping at 2.2 Hz. The low TE (0.01–0.02) and ES (≤ 0.31) for CT, FT and HopFreq support this. Thus, HopFreq was controlled adequately throughout.

Despite regulation of HopFreq in the present study, k_leg_ exhibited moderate reliability at 1.5 Hz and weak reliability at 2.2 and 3.0 Hz (ICC = 0.59–0.89) between inter-day test bouts. These reliability indexes were accompanied by strong consistency for k_ankle_ at 1.5 Hz only. Remaining k_joint_ variables demonstrated weak-to-moderate agreement between Day_1, Day_2 and Day_3 testing bouts. Between Day_3 and Day_3_Offset_, k_leg_ exhibited moderate reliability at 3.0 Hz but weak consistency at the other frequencies. The moderate index for k_leg_ at 3.0 Hz was accompanied by strong consistency for k_ankle_. Variables of k_knee_ and k_hip_ however, exhibited weak-to-moderate consistency throughout. Examination of kinematic and kinetic inputs (Tables 3 and 4) showed that limits in k_leg_ reliability occurred due to limits in ΔLeg_L_ reliability, while F_z max_ exhibited moderate-to-strong consistency. The present data also showed that limits in k_joint_ measurement agreement resulted from inconsistencies in M_joint max_ and Δϴ_joint_ at the highest hopping frequencies (2.2 and 3.0 Hz).

While the present study did not include a control condition, previous work in our lab examined the reliability of k_leg_ and k_joint_ measures in overground hopping [22]. This study employed the same methodology and used physically active participants consistent with the current work. Our work showed strong agreement (ICC = 0.95–0.98) for k_leg_ and k_ankle_ measures at different hopping frequencies [22]. In contrast, k_knee_ and k_hip_ exhibited weak-to-moderate agreement. Given our previous findings, that many stiffness variables showed weak-to-moderate agreement in the present study is surprising. Particularly since the primary function of the current inclined-SBS is to limit extraneous upper body movement to enhance landing and measurement consistency during SSC tasks [8,19].

It is understood that in biological measurement, observed scores are composed of a true score and additional error [42]. Weir [42] suggests that errors in measurement data result from instrumentation errors associated with equipment used, in addition to participant / tester error, modelling error and biological variability. In the context of the current work, instrumentation errors arise from equipment (force plate and video) use and protocol adherence between testing bouts. It is important to note that participants were instructed refrain from physical activity prior to data collection. Furthermore, force plate and video equipment were calibrated as per the manufacturer’s guidelines prior to each data collection period. In addition, equipment set-up and protocols used were consistent with published works showing strong reliability for k_leg_ and k_ankle_ during overground hopping [22]. Consequently, deteriorations in measurement consistency relative to published reports are not due to differences in testing protocols between studies.

Of additional concern to the reliability of k_leg_ and k_joint_ scores is error derived from the use of mathematical models. Whittlesey and Hammill [47] noted that models are sensitive to the number of model components. Thus, small errors present early in the modelling process will propagate by the end of the simulation. This suggests that modelling errors will increase as calculations progress from distal to proximal joints. As k_joint_ is the ratio of changes in M_joint_ and ϴ_joint_, it is clear that errors in M_joint_ (and k_knee_ and k_hip_) measures will increase as inverse dynamics analysis progresses from distal to proximal joints. It is important to consider that while these errors will influence k_joint_ consistency, they are unlikely to influence k_leg_ in the present work. In addition, as modelling procedures employed in the current work are consistent with previous works [22], it is unlikely that differences in measurement consistency between the current and previous works result from modelling errors.

Considering biological variability, our previous work on overground hopping noted the existence of k_leg_ as an attractor state [22] where participants are drawn to the manipulation of leg compression (seen by variable k_joint_ or ΔLeg_L_) to maintain a consistent k_leg_ under variable landing conditions [9,45]. These works also showed that stiffness variables were accompanied by mostly strong consistency for F_z max_ and ΔLeg_L_ variables. In the present study, it must be considered that while the reliability of F_z max_ was strong, the reliability of ΔLeg_L_ was weak-to-moderate across all testing bouts. As a result, the k_leg_ exhibited weak-to-moderate consistency throughout. It is therefore difficult to establish a consistent outcome variable (i.e. attractor state) that participants are trying to regulate. Furthermore, considering limits in the reliability of kinematic and kinetic variables measured, and the limited pattern to the nature of this reliability, we feel that our data does not support the notion that limits in reliability are due to kinematic variability to achieve consistent outcomes.

It is important to consider the novelty of the hopping task and its impact on the present data. Our previous work showed that familiarizing participants with testing procedures prior to data collection enhanced measurement reliability relative to earlier reports [23]. In the present study however, participants completed a familiarization session prior to data collection. Thus, acquainting participants with procedures did not benefit the consistency of hopping dynamics within the current SBS. One reason for this is likely the design of the current SBS. The design of the current SBS requires participants to adopt a flexed torso position while secured to it. This novel hopping position would alter the functional length of lower limb musculature compared to upright hopping, which will have impacted participants’ ability to achieve consistent hopping dynamics. In addition, the mass of the chair of the current SBS was 19.6 kg. The added mass may have made it difficult for participants to achieve consistency in their hopping patterns. This is partly supported by the work of Kramer et al. [48]. The authors demonstrated that participants achieved almost natural reactive jumps following this four-week period (i.e. 12 sessions). While the instructions regarding landing dynamics differed from the current study, the design of the lightweight sled (5 kg) allowed participants to adopt natural movement patterns [27,48]. Although the authors did not report reliability indices for variables measured, the changes seen (38% increase) following four weeks of training (i.e. familiarizing) suggest the performance of SSC tasks within an SBS to be a novel task requiring extensive participant familiarization. The practicalities of including multiple familiarization sessions prior to data collection must also be considered.

Considering the lack of reliability evident in kinematics, kinetics and stiffness variables measured, it is appropriate to accept the null hypothesis. Limiting extraneous upper body movement by using an SBS during single-leg hopping did not improve the reliability of k_leg_ and k_joint_ measures relative to previous reports on overground hopping. In fact, use of the current SBS reduced consistency of stiffness measures relative to previous reports on natural conditions.

## Conclusions

To summarize, the present data showed weak-to-moderate reliability for all stiffness measures during hopping using the current SBS. This was due largely to inconsistencies in leg compression (ΔLeg_L_). Thus, reducing extraneous upper body movement using the current SBS did not encourage consistent stiffness regulation. In fact, hopping within the current inclined-SBS had adverse effects on measurement consistency relative to published reports on overground hopping. Limits in the consistency of stiffness measures may be due to constraints imposed by restricting upper body movement, which introduced novelty to the single-leg hopping task. A single familiarization session was therefore inadequate to achieve consistent landing mechanics. The influence of sled design on the current findings must also be considered, with chair mass and orientation likely making it difficult for participants to achieve consistent hopping patterns. Researchers should consider these findings and endeavour to construct a light-weight system with a chair orientation that replicates the functional length of the muscle-tendon units driving the SSC task being analysed.
